# Ste11p MEKK signals through HOG, mating, calcineurin and PKC pathways to regulate the *FKS2 *gene

**DOI:** 10.1186/1471-2199-12-51

**Published:** 2011-11-24

**Authors:** Xiaoyan Wang, Mark A Sheff, David M Simpson, Elaine A Elion

**Affiliations:** 1 Department of Biological Chemistry and Molecular Pharmacology, Harvard Medical School, Boston, MA 02115, USA; 2 Protein Science Key Laboratory of the Ministry of Education, School of Medicine, Tsinghua University, Beijing 100084, PR China; 3 Martek Biosciences, Nautilus Court North, Boulder, CO 80301, USA

## Abstract

**Background:**

The *S. cerevisiae *MAPKKK Ste11p, a homologue of mammalian MEKK1, regulates three MAPK cascades for mating, invasive growth and osmotic stress and provides functions that are additive with the cell wall integrity pathway. Cell wall integrity requires the *FKS2 *gene that encodes a stress-induced alternative subunit of beta-1, 3 glucan synthase that is the target of echinocandin 1,3- beta glucan synthase inhibitors. The major signal transduction pathways that activate transcription of the *FKS2 *gene include the cell wall integrity and calcineurin pathways, and the Ste11p pathway.

**Results:**

Here it is shown that catalytically active Ste11p regulates *FKS2-lacZ *reporter genes through Ste12, calcineurin/Crz1p- and PKC pathways and the high osmolarity pathway. Ste11p stimulated the cell wall integrity MAPK Mpk1p (Erk5 homologue) and *FKS2 *independently of the mating pathway. Ste11p regulated *FKS2 *through all known and putative substrates: Pbs2p MAPKK, Ste7 MAPKK, Cmk2p calmodulin dependent kinase and Ptk2p kinase. Ste11p increased the expression level of Cmk2p through transcription-dependent and -independent mechanisms.

**Conclusions:**

The data suggest Ste11p regulates the *FKS2 *gene through all its known and putative downstream kinase substrates (Pbs2p, Ste7p, Cmk2p, and Ptk2p) and separately through Mpk1p MAPK. The patterns of control by Ste11p targets revealed novel functional linkages, cross-regulation, redundancy and compensation.

## Background

Ste11p encodes a mitogen activated protein kinase kinase kinase (MAPKKK) that activates conserved MAPK pathways controlling mating, high osmolarity glycerol (HOG), invasive growth and the *FKS2 *gene in the cell wall integrity pathway [1-3]. The catalytic domain of Ste11p is most homologous to mammalian MEKK1 [4]. During mating, high osmolarity growth and invasive growth, Ste11p is phosphorylated and activated by Ste20p, a p21 activated kinase that binds to Cdc42p. Once activated, Ste11p has the potential to phosphorylate and activate two MAPKKs, either Ste7p for mating and invasive growth pathways, or Pbs2p for the high osmolarity sensing pathway [5]. The activation of Ste7p leads to activation of Fus3p and Kss1p MAPKs that activate shared and unique transcription factors among other substrates [6,7]. Pbs2p activates Hog1p MAPK, which also activates transcription factors and other substrates [8,9].

Prior work suggests that Ste11p MAPKKK signals through MAPK Kss1p to positively regulate cell wall integrity [2]. This analysis revealed that Ste11p has functional redundancy with the Bck1p MAPKKK in the PKC pathway and can activate the expression of the *FKS2 *gene [2]. *FKS2 *encodes a stress induced beta-1,3 glucan synthase subunit similar to constitutively expressed *FKS1 *that is important for cell wall integrity under conditions of cell wall stress [1]. The Fks1p and Fks2p subunits of glucan synthase are major therapeutic targets of anti-fungal inhibitors and acquire resistance mutations during fungal infections in people [10,11]. *FKS2 *is expressed at low levels and is upregulated under conditions of stress, higher temperature, when *FKS1 *is mutated, reduced carbon source [1], and when glycosylation is disrupted [2].

The calcineurin and protein kinase C and mating pathways are the major signaling pathways that regulate *FKS2 *expression and cell wall integrity. Calcineurin (Cna1p catalytic subunit and a regulatory subunit Cnb1) is activated by increases in intracellular calcium by influx of extracellular calcium through a Mid1/Cch1-Ca^2+ ^channel [1,12,13]. Many of its responses are mediated through the transcription factor Crz1p. Calcineurin dephosphorylates Crz1p/Tcn1p, leading to nuclear localization that activates many genes [14]. During conditions of high extracellular calcium or pheromone, the calcineurin pathway signals Crz1p/Tcn1p to bind the *FKS2 *promoter at a calcineurin-dependent response element (CDRE) site within residues -928 to -706 [1,14]. During polarized growth, cell wall damage or temperature stress, the cell wall integrity pathway plasma membrane sensors signal through Rho1p to Pkc1p, which activates Bck1p MAPKKK to activate Mkk1/2p MAPKK, which signals Slt2p/Mpk1p MAPK and Mlp1p pseudokinase to activate several transcription factors. Slt2p/Mpk1p, its human homolog Erk5p, and Mlp1p activate *FKS2 *through the cell cycle transcription factor SBF (Swi4p/Swi6p) at a SCB consensus site at -385 to -391 [15]. There are three potential Ste12 TGAAACA binding sites starting at -894 to -899, but Ste12 has not been found to bind the *FKS2 *promoter in two independent CHIP studies. Mating pheromone induces *FKS2 *at late times [16], supporting the possibility that it is due to secondary events including activation of the calcineurin and PKC pathways from calcium influx and polarized growth.

One gene that is induced by Crz1p is *CMK2*, which encodes one of two redundant calmodulin dependent kinases, possibly an orthologue of human CaM kinase II [17,18]. Cmk2p prevents death of calcineurin-deficient cells under conditions of low calcium [19]. A genomic *in vitro *screen for kinase substrates suggests that Cmk2p could be a direct substrate of Ste11p [20], Ptk2p was the only other kinase substrate of Ste11p identified [20]. Ptk2p is a putative serine/threonine protein kinase involved in regulation of plasma membrane ATPase, spermine and spermidine transport [21-23] and stimulates Slt2p/Mpk1p phosphorylation [24]. Cmk2p is predicted to be phosphorylated by three different MAPK pathway kinases, Pbs2p, Fus3p and Slt2p/Mpk1p, and Ptk2p is predicted to be phosporylated by one of these three, Pbs2p (Additional File 1, Figure S1, MAPK pathway phosphorylation links to Cmk2p and Ptk2p defined *in vitro *by Ptacek *et al*., 2005 [20]), raising the possibility of cross regulation and functional redundancy.

Here we analyze how Ste11p regulates the *FKS2 *gene. Our results suggest that active Ste11p that is uncoupled from upstream control crosstalk to *FKS2 *through all four known possible kinase targets; through the HOG pathway MAPKK (Pbs2p), Cmk2p, and Ptk2p in addition to Ste7p MAPKK and stimulates the *FKS2 *gene independently of Ste12p. These findings reveal new functional links between Ste11p downstream targets to calcineurin and PKC pathways and illustrate a high level of signaling flexibility.

## Results

### Ste11p is required for basal Slt2p/Mpk1p activation

**Figure 1:**
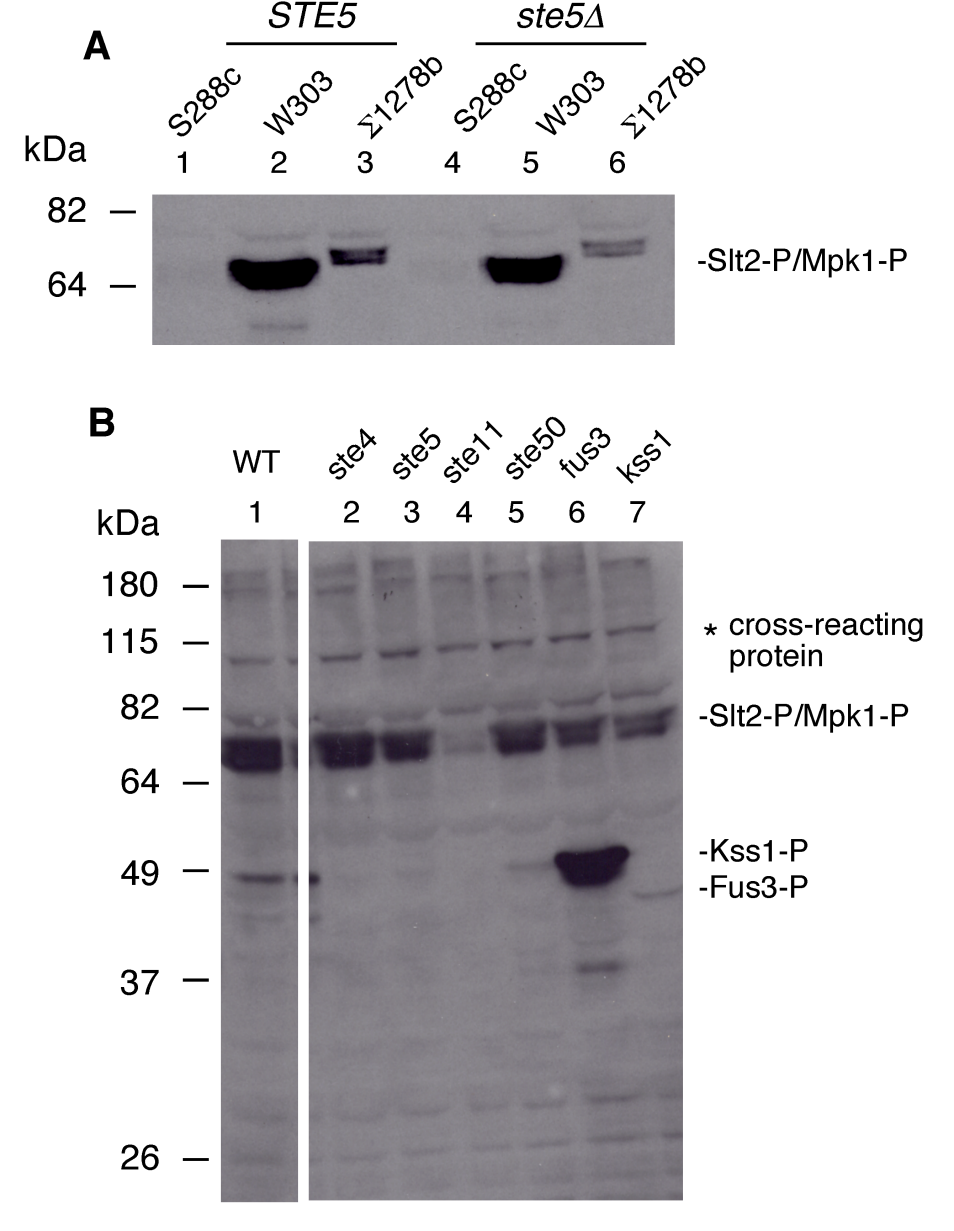
**Level of active Slt2p/Mpk1p, Fus3p and Kss1p detected with phospho-p42p44 antibody**. (A) Active Slt2p/Mpk1p in S288c, W303, ∑1278b backgrounds. (B) Active Slt2p/Mpk1p, Kss1p and Fus3p in S288c BY4741 and *ste4*Δ, *ste5*Δ, *ste11*Δ, *ste50*Δ, *fus3*Δ, *kss1*Δ null derivatives. Cells were exponentially grown in YEPD in A. and in SC selective medium with 2% dextrose in B. The data in Figure 1A are from a reprobing of a published immunoblot of active Kss1p and Fus3 (Supplemental Figure Two of Andersson *et al*., 2004 [25]).

Prior work indicated that Ste11p is functionally redundant with the Bck1p MAPKKK for cell integrity during vegetative growth and could basally activate the cell wall integrity target *FKS2 *gene using a well established *FKS2(-928 to +6)-lacZ *reporter gene [2]. To test the possibility that Ste11p regulates cell integrity through activation of the PKC pathway during vegetative growth, we assessed the basal level of active Mpk1p in three yeast strain backgrounds, S288c, W303a and ∑1278b by probing whole cell extracts with a mammalian phospho-p42p44 antibody that cross-reacts with *S. cerevisiae *Slt2p/Mpk1p [25]. We compared the level of active Slt2p/Mpk1p in exponentially dividing cells (Figure 1A, a longer exposure that also shows active Kss1p is in Additional file 2, Figure S2). The level and gel mobility of basal active Slt2p/Mpk1p varied tremendously. W303a and S288c had an equivalently sized short form of Slt2p/Mpk1p, whereas ∑1278b had a longer form. W303a had the highest basal level of active Slt2p/Mpk1p, whereas S288c had the lowest basal level, indicating variability at the level of pathway flux.

The basal level of active Slt2p/Mpk1p was clearly reduced in a *ste11Δ*, mutant (Figure 1B, S288c background shown). Less obvious partial reductions in the level of active Slt2p/Mpk1p were detected in *ste4Δ*, *ste5Δ*, *ste50Δ*, *fus3Δ*, *kss1Δ*, and *ste5Δ fus3Δ *strains (Figure 1A-1B, and data not shown). These observations suggested that Ste11p had a more critical role in regulating the PKC pathway than other mating pathway components.

### *STE11-4 *activates the *FKS2 *promoter through -928 to -706 and -706 to -540

**Figure 2:**
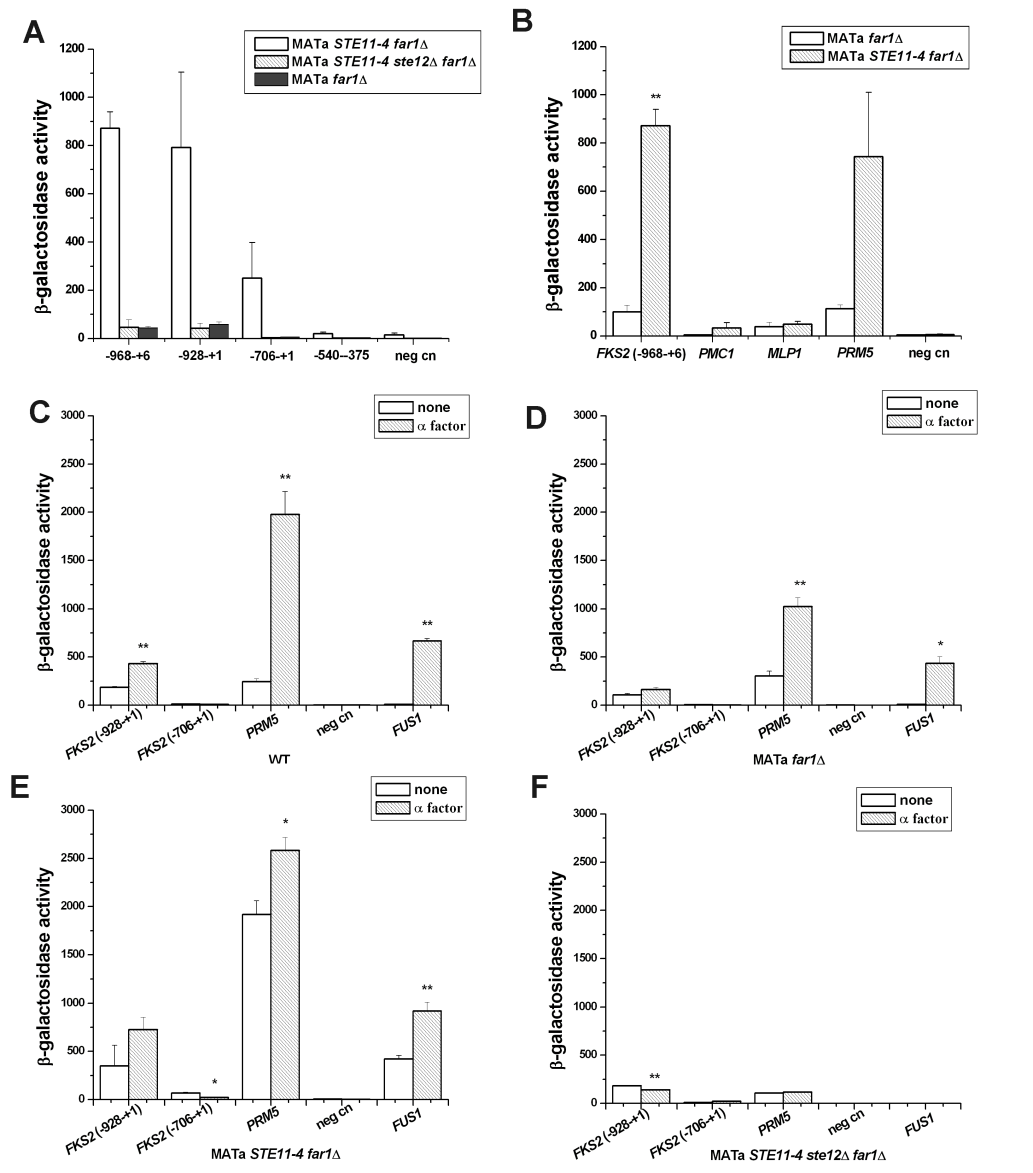
**Effect of *STE11-4 *and α factor on calcineurin pathway and cell wall integrity pathway reporter genes**. (A) Effect of *STE11-4 *and *ste12*Δ mutations on expression of different fragments of the *FKS2 *promoter fused to the *E. coli lac*Z gene. (B) Effect of *STE11-4 *on other promoter of calcinerin pathway and cell wall integrity pathway. (C) Effect of α factor in wild type strain. (D) Effect of α factor on promoter reporter genes in *far1*Δ strain. (E) Effect of *STE11-4 *and α factor on promoter reporter genes in *far1*Δ strain. (F) Effect of *STE11-4 *and α factor on promoter reporter genes in *far1*Δ *ste12*Δ strain. Data were expressed as mean ± SE of at least three independent experiments. Statistical significance was computed by the unpaired Student's *t *test. *p < 0.05, **p < 0.01.

The *FKS2 *promoter is complex with multiple promoter elements that include the calcineurin-dependent response element (CDRE), Swi4/Swi6 SBF element, glucose repression elements and potential pheromone response elements [1,14]. We examined how Ste11p regulates the *FKS2 *gene by comparing the ability of hyperactive *STE11-4 *to stimulate established *FKS2-lacZ *promoter truncations of the promoter: *FKS2*(-928+6)-*lacZ *(pDM5; [26]), *FKS2*(-928to+1)CYC1-*lacZ *[14], *FKS2*(-706+1)CYC1-*lacZ *[14] and *FKS2*(-540-375)CYC1-*lacZ *(p2052; [15]). The *FKS2 *-928 to +1 promoter has an SBF site at -385 CACGAAA-391 that binds a Swi4/Swi6/Mpk1 complex (assayed in YPD or supplemented SD [15]), the CDRE (calcineurin-dependent response element) within -762 to -705 (that includes a Crz1p -740 CAGTCGGTGGCTGTGCGCTTG-760 element that supports *LacZ *expression when present in 2 copies and assayed in synthetic medium containing 200 mM CaCl_2 _and ammonium chloride substituted for ammonium sulfate; [27]), a glucose repression element overlapping two putative Mig1p repressor consensus sites at -847 and -785 [15], and one of three Ste12p consensus binding sites, TGAAACA (-899-894). Consistent with prior work [2], *STE11-4 *activated both *FKS2 (-968 to +6)-CYC1-lacZ *and *(-928 to +1)-CYC1-lacZ *in 1X synthetic medium containing 2% dextrose and lacking uracil [(Figure 2A, B; negative control ("neg cn")] is pLGΔ-178 p*CYC1*-*lacZ*). *STE11-4 *also activated the *FKS2*(-706 to +1*)CYC1-lacZ *promoter that contains the SBF recognition site that is positively regulated by Mpk1p/Slt2p and Mlp1 (Figure 2A). *STE11-4 *activation was three-fold greater on the *FKS2 *-928 to +1 promoter than on -706 to +1, consistent with the presence of a putative Ste12p binding site and the CDRE element. *STE11-4 *failed to stimulate the *FKS2(-540 to -375)-lacZ *reporter that contains the SBF consensus site (-385 CACGAAA -391) that is regulated by Swi4p/Swi6p/Mpk1p/Mlp1p complexes and stimulated by temperature stress and congo red [15]. The absence of stimulation of the SBF site is consistent with *STE11-4 *and mating pheromone-induced inhibition of Cdc28 cyclin dependent kinase [28]. Thus, under our growth conditions, Ste11-4p positively regulated *FKS2 *reporter genes through at least two promoter elements, from -928 to -706 containing a Crz1p binding site, two potential Mig1 binding sites and 1 potential Ste12p binding site, and another element between -706 and -540, but did not stimulate transcription through the Mpk1p/Mpl1p responsive SBF site within -540 to -375.

### *STE11-4*, α factor, and Ste12 differentially regulate *FKS2-lacZ*

Mating pheromone stimulates *FKS2 *gene expression at late times, with optimal expression occurring 90 minutes after α factor addition in YEPD medium [16] and less obvious induction in synthetic medium [27]. α factor induction of genomic *FKS2 *mRNA is blocked by FK506 [16] and a *crz1Δ/tcn1Δ *mutation [16,27]. We compared the ability of α factor to stimulate *FKS2(-928 to +1)-lacZ *and *FKS2(-706 to +1)*-*lacZ *and two Ste12-dependent reporter genes *FUS1-lacZ *and *PRM5-lacZ*. In a wild type *STE11 *background, α factor addition caused only a modest 2-fold increase in expression of the *FKS2 *(-928 to +1)-*lacZ *reporter and no effect on the *FKS2 *(-706 to +1)-*lacZ *reporter. Much larger increases occurred for *PRM5-lacZ *(20-fold) and *FUS1-lacZ *(>100-fold) reporter genes that harbor multiple Ste12p consensus binding sites (Figure 2C). In wild type, *far1Δ*, and the *STE11-4 far1Δ *background (which has reduced levels of active Cln2p/Cdc28p compared to wild type [28]), α factor caused little increase in *FKS2 *(-928 to +1)-*lacZ *levels (i.e. 0.2-<2-fold). The *far1Δ *strain *(*which has increased basal levels of G1 cyclin dependent kinases compared to wild type [28]), had lowest basal levels of *FKS2(-928 to +1)-lacZ *and blocked α factor (Figure 2C,E). This contrasted up to >100 fold increases in *FUS1-lacZ *and *PRM5-lacZ *(Figure 2C-E).

By comparison, α factor stimulated the pDM5 *FKS2(-968 to +6)-lacZ *approximately 4-fold in a different wild type background [26]. Thus, regulation of reporter genes with -928 and -706 cut offs was relatively independent of α factor, consistent with only one Ste12p TGAAACA site very close to the 5'end of the -928 *FKS2-lacZ *constructs near the CDRE.

The absence of a strong α factor effect suggested that Ste12p might not be required for *STE11-4 *activation of *FKS2*. To determine whether the Ste12p transcription factor was required for Ste11p activation of *FKS2*, we tested the effect of a *ste12*Δ mutation in *STE11-4 far1Δ *strains treated or not with α factor compared with that in *no ste12Δ *mutation strains (Figure 2E, F). The *ste12Δ *null mutation blocked the ability of *STE11-4 *to activate both the *FKS2 *(-928 to +1)-*lacZ *and *FKS2 *(-706 to +1)-*lacZ *reporter genes similar to the blocks that occurred for *PRM5*-*lacZ *and *FUS1-lacZ *reporter genes. The effect was similar to that seen for α factor. Thus, Ste12p is critically important for *STE11-4 *to activate the *FKS2-lacZ *reporter genes, but its function is likely indirect.

### Ste12 stimulates the expression of *FKS2 *(-928 to +1)-*lacZ *but not *FKS2 *(-706 to +1)-*lacZ*

A *ste12Δ *mutation blocks the expression of pheromone response pathway genes that lead to activation of Ste11p, Ste7p and Fus3p and Kss1p MAPKs. Over expression of Ste12p can bypass positive and negative control by Fus3p and Kss1p MAPKs. We examined whether a *GAL1prom-STE12 *gene would increase the expression of the *FKS2 *reporter genes in the absence of calcineurin and mating pathway components. Cells were shifted from dextrose medium to raffinose medium and finally to galactose medium to induce expression of *GAL1prom-STE12 *(Materials and Methods). *FKS2 *gene expression is derepressed in poor carbon sources including raffinose and galactose, most likely through Snf1p kinase inhibition of Mig1p, which may bind consensus sites at -847 and -785 [14].

**Figure 3:**
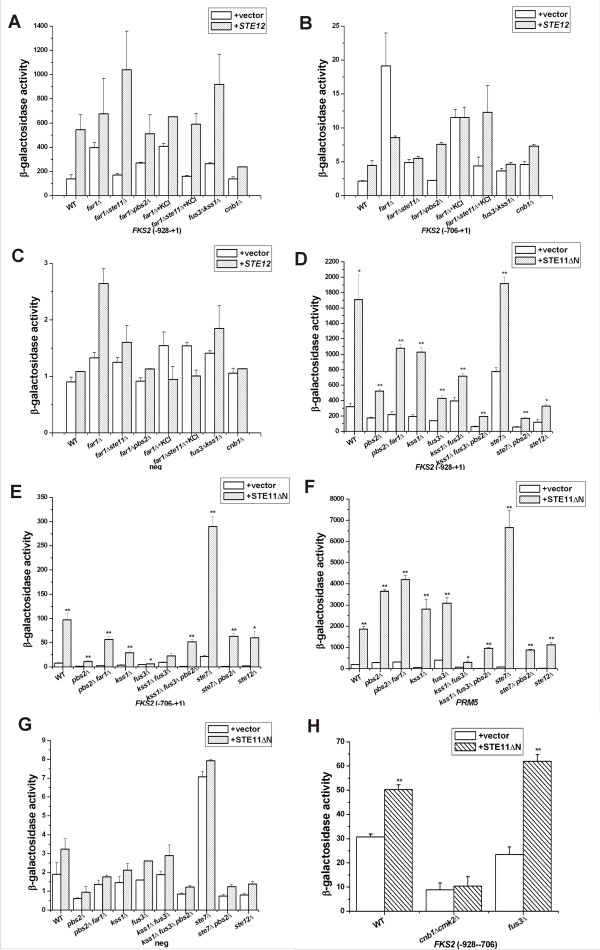
***STE12 *and *STE11ΔN *activation of *FKS2 *promoter in mating and HOG pathway mutants**. *GAL1prom-STE12 *over expressed in presence of *FKS2 *(-928 to +1)-*lacZ *(A), *FKS2 *(-706 to +1)-*lacZ *(B) and Δ-178 pCYC1-lacZ (C). *GAL1prom-STE11ΔN *over expressed in presence of *FKS2 *(-928 to +1)-lacZ (D), *FKS2 *(-706 to +1)-lacZ (E), *PRM5-lacZ *(F) and Δ-178 pCYC1-lacZ (G) and *FKS2*(-928 to -706)*-lacZ *(H). Strains were pregrown in raffinose- dextrose medium and then in galactose medium for 6 hours at 30°C. Data were expressed as mean ± SE of at least three independent experiments. Statistical significance was computed by the unpaired Student's *t *test. *p < 0.05, **p < 0.01.

Ste12p stimulated the expression of *FKS2 *(-928 to +1)-*lacZ *>5-fold in wild type, *ste11Δ*, *fus3Δ kss1Δ *and *far1Δ *strains, but not if the strain lacked the Cnb1 regulatory subunit of calcineurin (compare wild type and *cnb1Δ*, Figure 3A, C). In sharp contrast, *GAL1p-STE12 *did not activate the expression of *FKS2 *(-706 to +1)-*lacZ *to a great extent (Figure 3B, C), although *STE11-4 *was able to stimulate (Figure 2). Thus, Ste12p activated the *FKS2-lacZ *promoter by a mechanism involving the calcineurin pathway, suggesting it may mimic the late onset of activation by mating pheromone that leads to an increase in calcium influx that activates the calcineurin pathway CDRE element. In contrast, Ste11-4p activated the *FKS2 *promoter through the CDRE element as well as a second element within -706 to +1.

### Ste11ΔNp activates the *FKS2 *promoter from -928 to +1 through Pbs2p and Ste7p

It was unlikely that the block in expression of the -928 and -706 *FKS2*-*lacZ *constructs in the *STE11-4 ste12*Δ *far1*Δ strain was the result of a direct effect of Ste12p on *FKS2 *expression. However, the activity of Ste11-4p requires proteins that are regulated by Ste12p, such as Ste5p; for example, a dominant active *STE5hyp1 *gene also increased the level of active Mpk1 in anti-active MAPK westerns (data not shown). To bypass dependence on upstream regulators of Ste11p, we used a *STE11ΔN *allele (*pGAL1-STE11ΔN*) that lacks the N-terminal regulatory domain that represses the catalytic kinase domain and also binds Ste5p scaffold, Ste50p and Sho1p [29-31]. The *STE11ΔN *mutation largely, but not completely, bypasses a requirement for Ste20p phosphorylation to be active. Ste11ΔNp activates Kss1p and more weakly activates Fus3p due to the absence of Ste5p binding to link Ste7p to Fus3p [32], but can activate Pbs2p MAPKK down to Hog1p efficiently [33].

We tested dependence on Pbs2p and Ste7p MAPKKs that are activated by Ste11p. Strikingly, in a *pbs2*Δ strain, basal *FKS2 *(-928 to +1)-*lacZ *expression decreased and remained greatly decreased in the presence of *STE11ΔN *compared to the wild type strain. In contrast, a *ste7Δ *mutation had little effect on *pGAL-STE11ΔN *induced expression of *FKS2 *(-928 to +1)-*lacZ*. However, the level of *FKS2 *(-928 to +1)-*lacZ *was lower in a *ste7*Δ *pbs2*Δ double mutant than in a *pbs2*Δ single mutant (compare Figure 3D, G), revealing contribution from Ste7p. Thus, Ste11ΔNp regulates the *FKS2 *promoter gene through Pbs2p and Ste7p, but Pbs2p is more crucial.

### Fus3p activated *FKS2 *(-928 to +1)-*lacZ *expression whereas Kss1p inhibited

We tested whether Ste11p required Fus3p and Kss1p MAPK targets of Ste7p, to stimulate *FKS2 *(Figure 3D, G). Fus3p and Kss1p have many targets including Ste12p and its repressors Rst1p/Dig1p and Rst2p/Dig2p. In a *fus3*Δ strain, the basal level of the *FKS2 *(-928 to +1)-*lacZ *reporter gene decreased to the same degree as in a *ste12*Δ strain. Fus3p was required for *STE11ΔN *to activate *FKS2 *(-928 to +1)-*lacZ *expression; the block in the *fus3*Δ strain was similar to that of the *ste12*Δ mutant. In contrast, the *kss1Δ *mutation caused a modest decrease in expression on its own and, conversely, restored expression in the *fus3Δ *mutant background (Figure 3D, G compare *fus3 kss1 *with *fus3*). Therefore, Fus3p and Ste12p activated *FKS2 *(-928 to +1)-*lacZ *whereas Kss1p had more inhibitory effect than positive.

Ste11ΔNp activated the *FKS2 *gene via Pbs2p in the absence of Kss1p and Fus3p or Ste7p. A *pbs2*Δ mutation reduced the level of *STE11ΔN *activation of the *FKS2 *(-928 to +1)-*lacZ *reporter gene in a *kss1*Δ *fus3*Δ strain similar to in a *ste7*Δ (Figure 3D, G). Therefore, Ste11ΔNp regulates the *FKS2-lacZ *gene through both Ste7p MAPKK and Pbs2p MAPKK, with Pbs2p signaling being more significant and Ste7p positive signaling being dependent on Fus3p, Kss1p and Ste12p.

### Ste11ΔNp activates *FKS2 *(-706 to +1)-*lacZ *through Pbs2p and Fus3p and Kss1p, whereas Ste7p blocks signaling by Pbs2p

*STE11ΔN *was strictly dependent on Pbs2p and Fus3p/Kss1p to stimulate the *FKS2 *gene in the absence of the CDRE element. All three single deletions, *pbs2Δ*, *fus3Δ *and *kss1Δ*, decreased *FKS2(-706 to +1)-lacZ *expression with nearly complete blocks occurring with *pbs2Δ *and *fus3Δ *(Figure 3E, G). In sharp contrast, Ste7p had primarily negative function in regulating the *FKS2 *(-706 to +1)-*lacZ *reporter gene, with 3-fold increase in *lacZ *level occurring in the *ste7Δ *strain compared to wild type (Figure 3E). The increased signaling in the absence of Ste7p was primarily dependent on Pbs2p, and on Fus3p/Kss1p (Figure 3E, i.e. the level of *FKS2*(-706 to +1)-*lacZ *for *pbs2Δ ste7Δ *is equivalent to *ste12Δ *and wild type strains, whereas the *pbs2Δ fus3Δ kss1Δ *strain is even lower). Thus, we can define two separable branches of control that are inhibited by Ste7p, one through Pbs2p and another through Fus3p and Kss1p. Loss of Ste7p may increase the ability of Ste11ΔNp to signal through Pbs2p and may also confer catalytically inactive functions to Fus3p and Kss1p.

The relative contributions of Ste7p to Ste11ΔNp stimulation of the *FKS2*(-928 to +1)-*lacZ *and *FKS2*(-706 to +1)*-lacZ *reporters suggested Ste11p/Ste7p's major positive regulatory function was within the -928 to -706 region that responds to pheromone and its major negative regulatory function was between -706 to +1. The analysis with *STE11-4 *(Figure 2A) suggested a target region between -706 to -540 rather than the -385 CACGAAA-391 site that binds the SBF-Mpk1 complex [15].

### Ste11ΔNp exhibits little dependence on Bni1p and Ste20p

**Figure 4:**
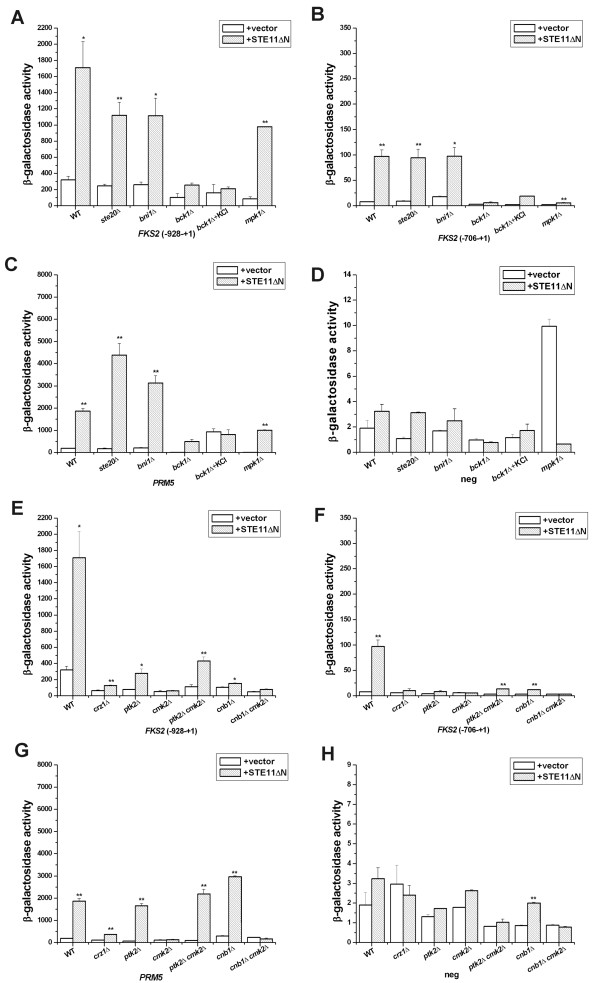
**Effect of mutations in PKC and calcineurin pathways and in *CMK2 *and *PTK2 *on Ste11ΔNp stimulation of reporter genes**. *STE11ΔN *activation of *FKS2 *(-928 to +1)-lacZ (A), *FKS2 *(-706 to +1)-lacZ (B), *PRM5*-lacZ (C) and Δ-178 pCYC1-lacZ (D) in cell wall integrity and filamentous pathway mutants. *STE11ΔN *activation of *FKS2 *(-928 to +1)-lacZ (E), *FKS2 *(-706 to +1)-lacZ (F), *PRM5-lacZ *(G) and Δ-178 pCYC1-lacZ (H) in calcineurin pathway mutants. Data were expressed as mean ± SE of at least three independent experiments. Statistical significance was computed by the unpaired Student's *t *test. *p < 0.05, **p < 0.01.

Bni1p and Ste20p are required for mating morphogenesis and pheromone activation of the PKC pathway and filamentation induced by Kss1p [1,33,34]. In both *bni1Δ *and *ste20*Δ strains, the basal level of the *FKS2 *(-928 to +1) reporter gene was the same as wild type strain and the level induced by Ste11ΔNp was reduced by ~25% compared to the wild type control (Figure 4A, D). Bni1p and Ste20p were required for full activation of *FKS2 *by Ste11ΔNp, but their contribution was much less than that of Ste7p, Fus3p, Kss1p and Pbs2p. There was no difference in expression of *FKS2 *(-706 to +1) reporter gene for wild type, *ste20*Δ and *bni1*Δ strains with *STE11ΔN *(Figure 4B, D). Thus, the *STE11ΔN *mutation largely bypasses a dependence on morphogenesis and filament formation to induce the *FKS2 *reporter genes.

### Ste11ΔNp activated the *FKS2 *promoter from -928 to +1 through cell wall integrity and calcineurin pathways

We determined whether Ste11ΔNp required the calcineurin and cell wall integrity pathways to stimulate *FKS2(-928 to +1)-lacZ *and *FKS2(-706 to +1)-lacZ *genes. Prior work demonstrated functional redundancy between Bck1p and Ste11p (based on synthetic sickness of *bck1Δ ste11Δ *double mutants [2]). In the course of this analysis, we found that a *bck1*Δ strain transformed with the *STE11ΔN *plasmid grew much better than *bck1*Δ cells without *STE11ΔN*, based on faster and greater density of growth in streak outs on SC selective plates (data not shown). This result supports functional redundancy between Bck1p and Ste11p [2] being mediated by targets of Ste11ΔNp.

The calcineurin pathway was required for Ste11ΔNp to induce expression of the *FKS2 *(-928 to +1)-*lacZ *reporter gene, as *lacZ *induction was blocked by mutations in calcineurin *cnb1Δ *and the target transcription factor *crz1Δ *(Figure 4E, H). Bck1p was also required for Ste11ΔNp to activate the *FKS2 *(-928 to +1) reporter gene, as was Mpk1p (Figure 4A, D). The *mpk1Δ *mutation reduced expression somewhat less than the *bck1Δ *mutation, presumably due to *MLP1 *functional redundancy [15]. The addition of an osmotic support, KCl, had no effect on the ability of Ste11ΔNp to support better growth or induce expression of the *FKS2 *(-928 to +1)-*lacZ *reporter gene, ruling out a trivial explanation of cell lysis for reduced expression in the *bck1Δ *strain or a block in signaling through the HOG pathway.

Ste11ΔN-induced expression of *FKS2 *(-706 to +1)-*lacZ *and *PRM5*-*lacZ *was also dependent upon both Cnb1p/Crz1p and Bck1p/Slt2p/Mpk1p pathways (Figure 4F, G, H), even though the *FKS2*(-706 to +1)*-lacZ *reporter lacks the calcineurin-dependent response element. The dependence on Bck1p and Slt2p/Mpk1p was as expected for both *FKS2 *(-706 to +1)-*lacZ *and *PRM5*-*lacZ *[1] whereas dependence on Cnb1p/Crz1p was unanticipated and revealed additional cross-regulation.

### Ste11ΔNp activated *FKS2 *(-928 to +1) and *FKS2*(-706 to +1) through Cmk2p and Ptk2p

We tested whether the two potential Ste11p substrates Cmk2p and Ptk2p [20] were required for *STE11ΔN *to activate *FKS2*-*lacZ *reporter genes. Loss of Cmk2p blocked the ability of *STE11ΔN *to activate the *FKS2 *(-928 to +1) reporter gene to a greater extent than *crz1Δ *or *cnb1Δ *mutations (Figure 4E, H). A *cnb1Δ cmk2Δ *double mutant was as equivalently blocked as a *cmk2Δ *single mutant. Thus, Cmk2p is important for Ste11ΔNp activation of *FKS2 *(-928 to +1)-*lacZ*.

Similar results were found for the *FKS2 *(-706 to +1)-*lacZ *reporter gene, except in this instance the inhibitory effects of the *cmk2Δ*, *cnb1Δ *and *crz1Δ *single mutations were more similar to each other (Figure 4F, H). Cmk2p and Cnb1p provided additive functions based on a greater block in *cmk2Δ cnb1Δ *double mutant than either single mutant. Moreover, dependence on Cmk2p and Ptk2p was also apparent using the *PRM5*-*lacZ *reporter gene that senses the PKC pathway (Figure 4G, H). Cmk2p was more critical than Cnb1p and clearly provided additive functions with Cnb1p as the introduction of a *cmk2Δ *mutation abolished residual signaling in the *cnb1Δ *strain. Therefore, Cmk2p was essential for Ste11ΔNp to signal to calcineurin and PKC pathway reporter genes.

The galactose-induced *pGAL1-STE11ΔN *gene had a ~2-fold stimulatory effect on a *FKS2*(-928 to -706)*-lacZ *reporter gene that includes the calcineurin CDRE and the Mig1p glucose repression element in wild type (2-fold) and *fus3Δ *(2.5-fold) backgrounds (Figure 3H). This fold-effect was similar to that of α factor on the (-928 to +1) construct. A *cnb1Δ cmk2Δ *double mutation reduced basal expression of *FKS2*(-928 to -706)*-lacZ *3-fold and completely blocked *STE11-4*-induced expression, confirming that expression required the calcineurin CDRE (Figure 3H). The larger fold-increases with *FKS2*(-928 to +1)-*lacZ *and *FKS2*(-706 to +1)-*lacZ *(Figure 3D, E) supports the likelihood that Ste11p regulates a *FKS2 *promoter region that lies within -706 to -540 (Figure 1A).

Remarkably, Ptk2p was also required for Ste11ΔNp to induce *FKS2 *(-928 to +1)-*lacZ *and *FKS2 *(-706 to +1)-*lacZ *(Figure 4E, F, H), and provided nearly as significant positive control as Cmk2p. Ptk2p and Cmk2p did not provide additive functions, based on no greater block in a *cmk2*Δ *ptk2*Δ double mutant than either single mutant (Figure 4E, F, H). The level of expression in the *cmk2*Δ *ptk2*Δ double mutant was similar to the *ptk2*Δ single mutant, which was greater than the *cmk2*Δ single mutant (Figure 4E, F, H). Ste11ΔNp induced *FKS2 *(-928 to +1)-*lacZ *expression in the *cmk2*Δ *ptk2*Δ strain to a greater degree than in the *cmk2*Δ single mutant suggesting Ptk2p provides both positive and negative control (Figure 4E). The *cmk2*Δ and *ptk2*Δ mutations each completely blocked Ste11ΔNp induction of the *FKS2 *(-706 to +1)-*lacZ *reporter that lacks the calcineurin CDRE (Figure 4F). Therefore, Cmk2p and Ptk2p are both required for Ste11ΔNp to signal to calcineurin and PKC pathway reporter genes, but Cmk2p plays a greater role than Ptk2p.

### *STE11ΔN *increased *PRM5 *mRNA and *FKS2 *mRNA

**Figure 5:**
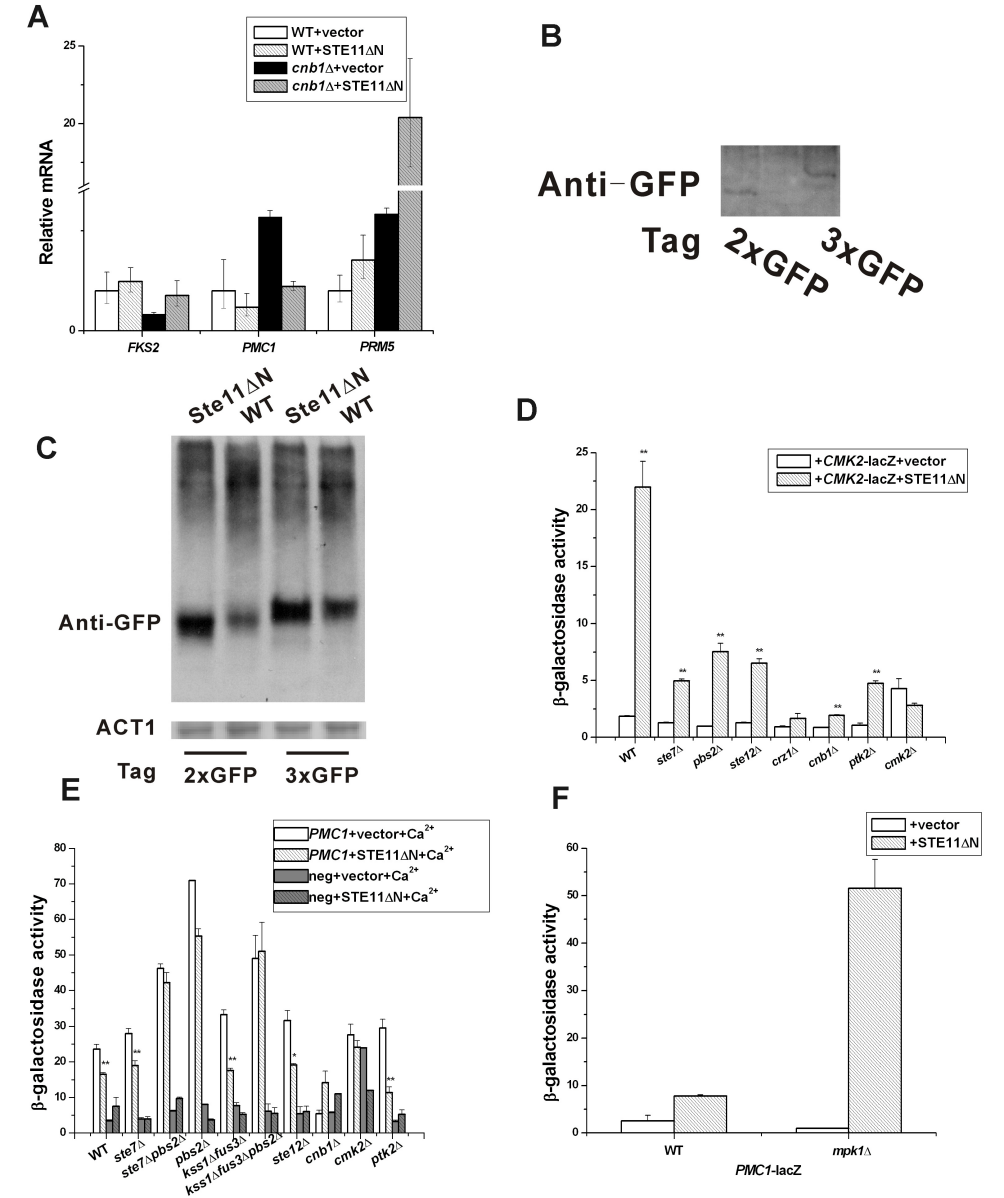
**Effect of Ste11ΔNp on Cmk2p, *CMK2 *and *PMC1 *reporter gene**. (A) Effect of *STE11ΔN *over expression on *FKS2*, *PMC1 *and *PRM5 *mRNAs. Cells were grown as in Figure 4. (B) Genomic *CMK2 *with triple and double *GFP *confirmed by western blotting. (C) Electrophoresis mobility and abundance change of Cmk2-GFP induced by *STE11ΔN*. (D) *CMK2*-*lacZ *activation by *STE11ΔN*. (E) Lack of effect of *STE11ΔN *on *PMC1*-*lacZ *with Ca^2+^. (F) *STE11ΔN *stimulates *PMC1*-*lacZ *in a *mpk1*Δ mutant. Data were expressed as mean ± SE of at least three independent experiments. Statistical significance was computed by the unpaired Student's *t *test. *p < 0.05, **p < 0.01.

We tested whether Ste11ΔNp increased *FKS2 *and *PRM5 *mRNAs in wild type and *cnb1*Δ strains. The mRNA levels for *FKS2*, *PRM5*, *PMC1 *and *ACT1 *were assessed by cDNA synthesis and real-time PCR using *ACT1 *as a normalization control. *FKS2 *mRNA increased in the presence of *STE11ΔN*, but a much larger increase was detected for *PRM5*, and no increase was detected for *PMC1 *mRNA (Figure 5A). The effect on *FKS2 *mRNA was less than what we detected with the *FKS2*-*lacZ *reporter and may reflect posttranscriptional effects or the fact that the promoter is complex with multiple signaling elements less easily distinguished than by using the reporter genes. A *cnb1*Δ mutation decreased the level of *FKS2 *and *PRM5 *mRNAs as expected but had no effect on basal mRNA of *PMC1*. Ste11ΔNp still increased *FKS2 *and *PRM5 *mRNAs in the *cnb1*Δ mutant, consistent with a calcineurin-independent pathway that could involve the PKC pathway.

### Ste11ΔNp increased the abundance of Cmk2p

The observation that Ste11ΔNp activation was blocked in *cmk2*Δ strains (Figure 4E, H) supported the possibility that Cmk2p might be an *in vivo *substrate of Ste11p [20]. To look for a posttranslational effect of Ste11p on Cmk2p, we determined whether Ste11ΔNp influenced the mobility of Cmk2p in a native polyacrylamide gel mobility experiment. Triple and double GFP tags were inserted at the carboxyl-terminal end of *CMK2 *which were confirmed by PCR (data not shown) and western blotting (Figure 5B). Immunoblot analysis of whole cell extracts revealed that the abundance of Cmk2p was greater in cells that express Ste11ΔNp (Figure 5C). This finding was consistent with an effect of Ste11p on the *CMK2 *promoter that is controlled by Crz1p and a posttranscriptional effect on the level of Cmk2p that could arise from feedback stimulation of its own expression [13]. A comparison of the mobility of *CMK2*-3XGFP and *CMK2*-2XGFP in wild type and Ste11ΔNp cells also suggested a faster mobility in the presence of Ste11ΔNp, which would be consistent with a posttranslational modification (Figure 5C). These data were consistent with the possibility that Ste11p regulated Cmk2p directly.

### Ste11ΔNp activated *CMK2-lacZ *through Cmk2p

The calcium signal is transmitted to both Cnb1p and Cmk2p, but only Cnb1p appears to activate Crz1p [19]. Calcium activation of a *CMK2-lacZ *reporter is blocked by a *crz1*Δ *cmk2*Δ double mutation but not by a *cmk2*Δ single mutation, indicating that Crz1p activates the *CMK2-lacZ *gene independently of Cmk2p [19]. To test for an effect of Ste11ΔNp on the *CMK2 *promoter that might be mediated through its effects on Cmk2p protein, we measured *CMK2-lacZ *expression in wild type and *cmk2*Δ strains. The *CMK2-lacZ *reporter is stimulated by 200 mM CaCl_2 _in the medium in a Crz1- and FK506-dependent manner [19]. Basal and Ste11ΔNp induction of *CMK2-lacZ *were blocked by *cnb1*Δ and *crz1*Δ single mutations (Figure 5D). Strikingly, Cmk2p was also required for Ste11ΔNp to activate the *CMK2-lacZ *reporter gene, suggesting feedback control. The *cmk2*Δ mutation caused a large block in Ste11ΔNp induction of *CMK2*-*lacZ *expression compared with the wild type *CMK2 *strain. Ste7p and Ste12p were also required for Ste11ΔNp to efficiently activate the *CMK2 *promoter as was Ptk2p (Figure 5D). Therefore, Ste11ΔNp uses multi-pathway crosstalk to regulate the *CMK2 *promoter. The strict dependence on Cmk2p suggests Ste11p activates the level or activity of Cmk2p protein, which then activates the *CMK2 *promoter through positive feedback.

### Ste11ΔNp activated morphogenesis in *cmk2*Δ strain

Ste11p and its known targets Ste7p and Pbs2p positively regulate morphogenesis. To determine whether Cmk2p had a role in morphogenesis, we counted the number of polarized and nonpolarized cells that were enlarged in wild type and *cmk2*Δ cells with and without Ste11ΔNp. Interestingly, in the *cmk2*Δ strain lacking Ste11ΔNp, only 2.1% cells displayed an enlarged morphology compared to 9.9% of wild type cells grown in YEPD medium (Table 1). These findings supported a role for Cmk2p in the control of morphogenesis. As expected, wild type cells expressing Ste11ΔNp grew bigger than control. In the *cmk2*Δ strain, fewer cells enlarged with Ste11ΔNp. However, Ste11ΔNp was still able to induce cell enlargement in the *cmk2*Δ strain. Collectively, these findings revealed that Cmk2p was important for morphogenesis and that Ste11ΔNp retained a strong ability to activate morphogenesis independent of Cmk2p, presumably through activation of one or more downstream targets (e.g. Pbs2p, Ste7p, Ptk2p, and Ste12p indirectly).

**Table 1:** Percentage of cells with enlarged morphology in wild type strain and *cmk2*Δ strains expressing Ste11ΔNp.

Strains	Enlarged morphology/total
WT+vector	9.9%
*cmk2Δ+*vector	2.1%
	
WT+*STE11ΔN*	8.5%
*cmk2Δ+STE11ΔN*	8.6%

### Ste11ΔNp also required Pbs2p, Ste7p and Cmk2p to activate a *PRM5 *reporter gene

To confirm the validity of our findings with *FKS2*, we examined the effects of mutations on *PRM5*, a second gene that senses both PKC pathway and mating pathway inputs (Figure 4C, D). Ste11ΔNp induction of expression of the *PRM5*-*lacZ *reporter gene was blocked by *fus3Δ kss1Δ *and *ste12Δ *mutations, but not by *fus3Δ *and *kss1Δ *single mutations (Figure 3F, G) as expected. In contrast, *ste7Δ *and *pbs2Δ *single mutations had no effect but Ste11ΔNp was blocked by the *ste7Δpbs2Δ *double mutation (Figure 3F, G). Thus, Pbs2p and Ste7p are both equivalently required for Ste11ΔNp to signal to *PRM5-lacZ*, a second PKC pathway reporter gene.

Among the various calcineurin pathway mutant strains examined, the *cmk2*Δ mutation caused the biggest block in Ste11ΔNp activation of *PRM5-lacZ*. A strong block occurred with a *crz1Δ *mutation, but only a partial decreased occurred for *cnb1Δ *and *ptk2Δ *mutations. The overall pattern of effects was similar to the *FKS2 *(-706 to +1) reporter gene, with Ptk2p being required for Cmk2p to regulate the *PRM5-lacZ *reporter (Figure 4G, H). The pattern of effects of mutations in *CMK2*, *PTK2*, *CNB1 *and *CRZ1 *resembled that found for *FKS2 *(-706 to +1)*-lacZ *reporter, which primarily senses the PKC pathway. Collectively, these findings supported Ste11p crosstalk of the PKC pathway as observed with the *FKS2-lacZ *gene and dependence on Pbs2p, Ste7p, Cmk2p and Ptk2p.

### Slt2p/Mpk1p blocked Ste11ΔNp activation of the *PMC1-lacZ *reporter gene

Since Ste11p crosstalked with downstream targets of the calcineurin pathway, we also tested *PMC1-lacZ*, another reporter gene of this pathway whose expression is stimulated by Ca^2+ ^[26]. When mutant strains were treated with Ca^2+^, *PMC1-lacZ *expression increased as reported (Figure 5E). However, Ste11ΔNp did not significantly increase *PMC1-lacZ *expression (Figure 5F). Instead, we found unexpectedly that Ste11ΔNp increased the level of *PMC1-lacZ *expression 5-fold in a strain lacking Mpk1p MAPK (Figure 5F; *MPK1 *and *mpk1Δ *strains grown in YEPD medium). This interesting result revealed that Slt2p/Mpk1p normally suppresses Ste11ΔNp activation of *PMC1-lacZ *expression. We examined the level of *PMC1 *mRNA by RT-qPCR and found that expression of Ste11ΔNp decreased *PMC1 *mRNA levels in both wild type and *cnb1*Δ strains (Figure 5A), which would be consistent with the ability of Ste11ΔNp to increase the level of active Slt2p/Mpk1p. Thus, Slt2p/Mpk1p may prevent Ste11p cross-regulation of the calcineurin pathway and feedback inhibit at some step upon activation by Ste11p.

## Discussion

**Figure 6:**
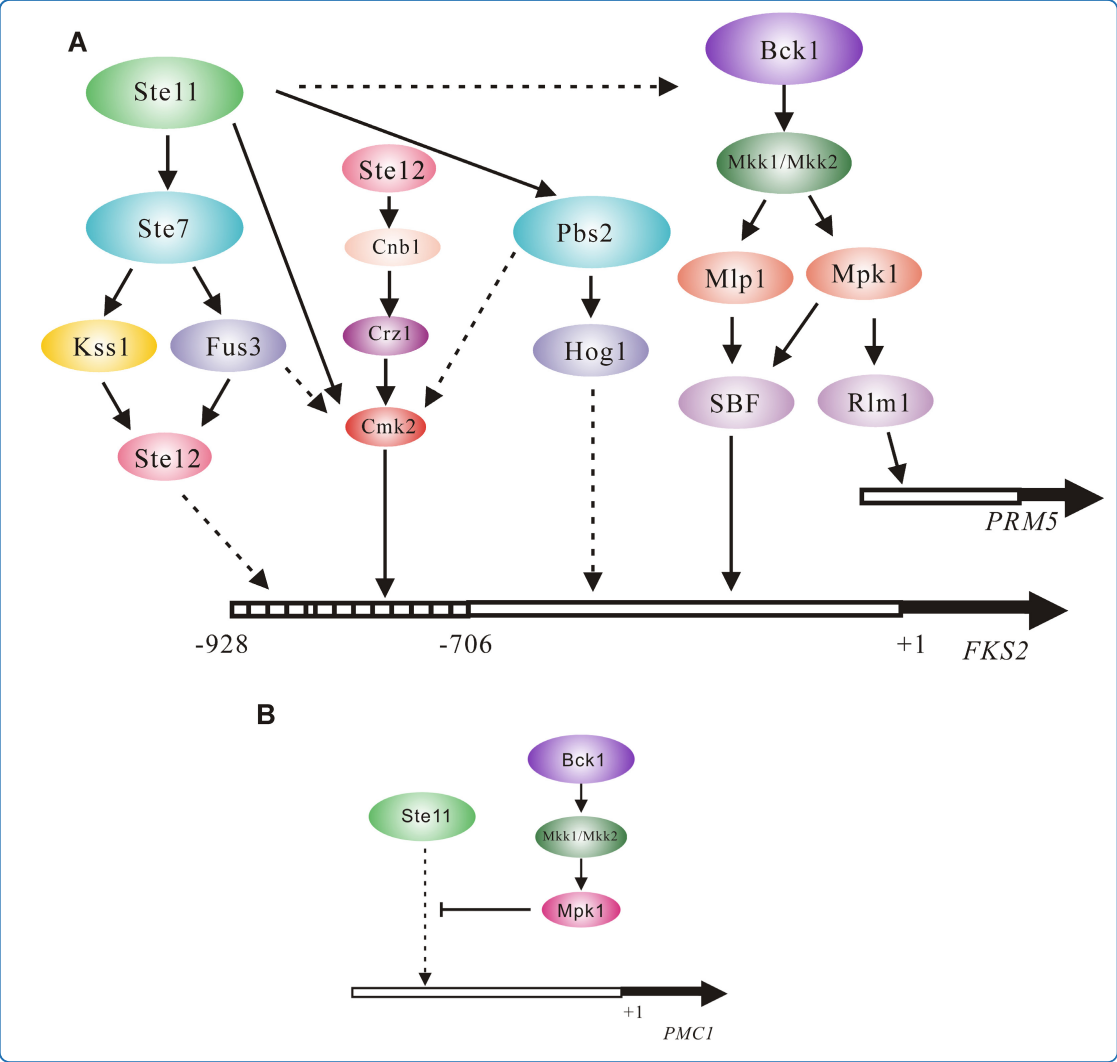
**Summary of Ste11p crosstalk through kinase targets to *FKS2***. (A) Ste11p crosstalks with mating, calcineurin, HOG, and cell wall integrity pathways. (B) Slt2p/Mpk1p may inhibit Ste11p activation of *PMC1*.

The activation of the calcineurin, cell wall integrity and high osmolarity pathways in *S. cerevisiae *and other fungi is important for cell survival and response to cell wall damage and other stimuli including calcium influx, polarized growth, hypo-osmolarity, pheromone, heat shock and perturbation of the actin cytoskeleton [1,35]. *FKS2 *is important for survival under stress conditions linked to the calcinerin and PKC pathways [1,14,15]. Prior analysis had suggested a potential link between mating pathway activation and the calcineurin and cell wall integrity pathways, but the means by which this signaling occurs was not fully understood [1,36]. We discovered that Ste11ΔNp crosstalks with cell wall integrity and calcineurin pathways through Pbs2p MAPKK, and two putative kinase targets Cmk2p and Ptk2p, with separable contribution from Ste7p MAPKK, Fus3p and Kss1p MAPKs. Support for the relevance of our findings is the logical pattern of effects of mutations in the genes encoding these protein kinases, and in calcineurin and cell wall integrity components (Additional File 1, Figure S1; Figure 6). Moreover, similar patterns of control were found for other physiologically relevant promoters, including *PRM5 *and *CMK2*. Additional support comes from the observation that the activation of *FKS2 *reporter genes by Ste11p did not require upstream activators or Ste20p or Bni1p-mediated morphogenesis, and the finding that activation of Mpk1p kinase (Figure 1) was more dependent on Ste11p than it was on mating pathway components such as Ste5p and Ste2p.

The data hint at the existence of a distinct promoter element within the *FKS2 *(-706 to +1) promoter region that is regulated by Ste11p, but not by Ste12p. *STE11-4 *did not activate a *FKS2*(-540 to -375)*-lacZ *reporter that strictly overlaps the Mpk1p regulated SBF element (Figure 2A), but could stimulate *FKS2*(-706 to +1)*-lacZ *and Mpk1p kinase activity. The Ste11p-inducible promoter element operates independently of Ste12p, because Ste12p could not activate *FKS2*(-706 to +1)*-lacZ *although it could activate *FKS2*(-928 to +1)*-lacZ *with the CDRE. Collectively, these observations suggest the Ste11p- inducible element overlaps the -706 to -540 region.

Multiple lines of evidence support a functional link between Ste11p and Cmk2p and the calcineurin pathway (Figure 6). While support for direct physical interaction between Cmk2p and Ste11p comes from the prior *in vitro *analysis [17], there has been no functional connection between these protein kinases. Four lines of evidence support Cmk2p being positively regulated by Ste11p. First, the effect of Ste11ΔNp on *FKS2 *expression was almost blocked without Cmk2p; Ste11ΔNp was functionally dependent on Cmk2p. Second, the *cmk2*Δ mutation caused a larger effect block of Ste11ΔNp stimulation of *PRM5 *expression that senses the PKC pathway than *crz1 *and *cnb1 *mutations (Figure 4G), supporting the likelihood that Cmk2p is a substrate of Ste11ΔNp. Third, there is a hint of a shift in the mobility of Cmk2-GFP when Ste11ΔNp is expressed (Figure 5C), supporting the possibility of posttranslational modification by phosphorylation. Fourth, Ste11ΔNp clearly increased the abundance of Cmk2-GFP (Figure 5C) and increased the expression of *CMK2-lacZ *in a manner dependent on Cmk2p (Figure 5D), consistent with the possibility of greater Cmk2p activity. These observations support direct positive control of Cmk2p by Ste11p phosphorylation, although more work is needed.

*In vitro *analysis suggests Cmk2p may be a target of phosphorylation by a number of kinases including Ste11p, Fus3p and Pbs2p and a central node in cellular control [20]. Cmk2p was the substrate of Ste11p, Pbs2p, Fus3p, Cka1p, Kns1p, Pho85p, Pho85-Pcll9, Slt2p/Mpk1p, Pkh2p, Tpk2p, Tpk3p, Ygl059w and Yol128c. Thus, the effects of *fus3Δ *and *pbs2Δ *mutations on expression of the various reporter genes in our study may be partly due to loss of regulation of Cmk2p by Fus3p and Pbs2p. The expression levels of *FKS2 *(-928 to +1)*-lacZ *and *FKS2 *(-706 to +1)*-lacZ *reporter genes were equivalently reduced to the same degree by the *pbs2*Δ or *fus3*Δ mutations. It is curious that a *ste7Δ *mutation had little effect since Ste7p activates Fus3p. However, Ste7p contribution could be detected in the *ste7*Δ *pbs2*Δ double mutant strain with Ste11ΔNp; *FKS2 *expression was 3-fold lower than in *pbs2*Δ or *fus3*Δ single mutant strains. Perhaps without its regulatory domain, Ste11p preferentially phosphorylates Pbs2p over Ste7p; this could provide an explanation for the lesser effect in a *ste7*Δ strain. An alternative explanation is that loss of Ste7p leads to an increase in Ste11ΔNp pathway flux through Pbs2p. In fission yeast, Cmk2p is phosphorylated in response to oxidative stress [37], supporting the possibility that Ste11p regulates Cmk2p in part through Pbs2p. Further work is needed to understand how Cmk2p and Ptk2p regulate theA *FKS2 *gene. Ptacek *et al*., 2005 identified 9 *in vitro *substrates for Cmk2p and 194 for Ptk2p [20]. Remarkably, four of these substrates overlap, supporting functional redundancy between Cmk2p and Ptk2p.

Our analysis reveals a possible feedback loop whereby Cmk2p activates its own expression through calcineurin (Cnb1). *CMK2-lacZ *expression was also partially dependent on Ste7p and Ste12p. Because Ste12p is a transcription factor, there are multiple genes through which it could regulate *CMK2-lacZ*. However, one gene could be *FUS3*, since Fus3p is both activated by Ste7p and may directly regulate Cmk2p [20]. The multi-pathway crosstalk through the *CMK2 *promoter that requires Cmk2p could reflect a cooperative activity of one or more transcription factors. Potential shared physical linkages between Cmk2p, calmodulin and calcineurin (Cmk2-Cmd1, [38-40]; Cmd1-Cna1p and Cmd1-Cnb1p, [38,39,41]), raises the possibility of a direct link between Cmk2p and calcineurin signaling to Crz1p.

An unexpected interesting observation was that Slt2p/Mpk1p inhibits the ability of Ste11p to regulate a calcineurin pathway gene, *PMC1*. In the W303a background, *PMC1 *expression could be activated in Ca^2+ ^medium (Figure 5E), but there was only modest activation by Ste11ΔNp (Figure 5E, F). However, an *mpk1Δ *mutation liberated Ste11ΔNp to increase *PMC1 *expression, even though it greatly decreased basal expression of *PMC1 *(Figure 5F). Therefore, Slt2p/Mpk1p inhibited Ste11ΔNp activation of *PMC1 *expression. One hypothesis is that Slt2p/Mpk1p may inhibit certain proteins in the calcineurin pathway that influence Ste11p function. One possible link is Cmk2p, which is also a putative *in vitro *target of Slt2p/Mpk1p [17]. Further analysis is required to understand how Slt2p/Mpk1p selectively blocks the ability of Ste11p to activate *PMC1-lacZ *and why this level of control is the reverse of what was detected for the *FKS2 *and *PRM5 *reporter genes that also sense calcineurin pathway and cell wall integrity pathways.

Our analysis suggests that in *S. cerevisiae*, there is much fluidity in pathway crosstalk between MAPK pathways. It was quite surprising to find that all possible Ste11p kinase targets were required for the full effect of Ste11ΔNp on activating various reporter genes. These findings suggest significant compensation and redundancy at the level of control and support functional relationships among the many targets of Ste11p.

The study also reveals that a transcription factor (i.e. Ste12p) can strongly regulate the expression of a gene through indirect means. Prior CHIP studies failed to find evidence of Ste12p binding to *FKS2 *[42,43] and α factor induction of *FKS2 *mRNA is completely blocked by FK506 and *crz1Δ *[16,27]. It has been suggested that mating pheromone stimulates the *FKS2 *gene indirectly through effects on calcium influx [14,27]. However, multiple pathways are activated by α factor mating pheromone and Ste12p has the capacity to bind to multiple transcription factors to regulate distinct pathways. Ste12p and Ste11ΔNp stimulated *FKS2*(-928 to +1)*-lacZ *reporter harboring the CDRE plus one of three Ste12p consensus TGAAACA sites (-1374GTGAAACA-1367 -1198CTGAAACA-1191 -901TTGAAACA-894), but stimulation was largely blocked by a calcineurin regulatory subunit *cnb1Δ *mutation. Thus, Ste12p is critically important for activation of *FKS2*, but its function is indirect within the promoter region examined in this study.

## Conclusions

Our results lead us to several conclusions: (1) Ste11p crosstalks with calcineurin and cell wall integrity pathways independently of the mating pathway. (2) Ste11p crosstalks through all known or potential downstream kinase targets, Ste7p, Pbs2p, Cmk2p and Ptk2p, to regulate calcineurin and PKC pathway reporter genes, *FKS2*, *PRM5*, and *CMK2*, (3) Cmk2p is likely to be a direct substrate of Ste11p in the calcineurin pathway *in vivo *based on functional dependence, (4) Ste11p increases the abundance of Cmk2p in part through a positive effect on the *CMK2 *gene promoter which is dependent on Cmk2p. An additional unexpected observation is that Slt2p/Mpk1p may prevent Ste11p from stimulating *PMC1 *expression, which could arise through Ste11p activation of Slt2p/Mpk1p. A summary of these observations is shown in Figure 6. Ste12p is important but its role is indirect.

## Methods

### Media, Plasmids, and Strains

Yeast extract/peptone/dextrose (YPD) and synthetic complete (SC) media with dextrose, raffinose or galactose were made according to standard laboratory methods. Yeast strains and plasmids used in this study are described in Table 2. Yeast strains are isogenic to W303a or Sigma or S288c strains. All yeast deletion strains derivatives were made either with published deletion plasmids or through PCR oligonucleotide cassette disruption with pFA6a-3xGFP-KAN as described [44]. EY957 (W303a *MATa bar1Δ *background) was engineered to harbor C-terminally tagged *CMK2 *using pFA6a-3xGFP-KAN following the experimental approach in [44]. Two *CMK2-GFP-KAN *integrants were recovered, one with *3xGFP-KAN *and the other with *2xGFP-KAN*.

**Table 2:** Yeast strains and plasmids used in this study.

Strain	Relevant genotype	Source or reference
Isogenic to W303		

EYL682	*MATa mpk1Δ::TRP1 ura3-1 trp1-1 leu2-3,112 ade2-1 ade3-1 his3 can1-100 lys2 (Gal+)*	Elion lab collection
EYL780	*MATa bar1Δ far1Δ ste11-4 ste12Δ::URA3 trp1-1 his3Δ200 leu2-3,112 ade2-1 ura3-1 can1-100 (GAL+) FUS1p-HIS3::lys2*	Elion lab collection
EY957	*MATa bar1Δ trp1-1 his3-11,15 leu2-3,112 ade2-1 ura3-1 can1-100 (GAL+)*	Elion lab collection
EY1298	*MATa bar1Δ far1Δ ste11-4 trp1-1 his3Δ200 leu2-3,112 ade2-1 ura3-1 can1-100 (GAL+) FUS1p-HIS3::lys2*	Elion lab collection
EYL4661	*MATa bar1Δ trp1-1 his3-11,15 leu2-3,112 ade2-1 ura3-1 can1-100 (GAL+) ste11Δ::Hygro*	Elion lab collection
EYL5328	*MATa bar1Δ trp1-1 his3-11,15 leu2-3,112 ade2-1 ura3-1 can1-100 (GAL+) pbs2Δ::Hygro*	This study
EYL5329	*MATa bar1Δ trp1-1 his3-11,15 leu2-3,112 ade2-1 ura3-1 can1-100 (GAL+) kss1Δ::HIS3 fus3Δ::Hygro pbs2Δ::Kan*	This study
EYL5330	*MATa bar1Δ trp1-1 his3-11,15 leu2-3,112 ade2-1 ura3-1 can1-100 (GAL+) ste12Δ::Hygro*	This study
EYL5331	*MATa bar1Δ trp1-1 his3-11,15 leu2-3,112 ade2-1 ura3-1 can1-100 (GAL+) bni1Δ::Hygro*	This study
EYL5332	*MATa bar1Δ trp1-1 his3-11,15 leu2-3,112 ade2-1 ura3-1 can1-100 (GAL+) ste7Δ::Hygro pbs2Δ::Kan*	This study
EYL5333	*MATa bar1Δ trp1-1 his3-11,15 leu2-3,112 ade2-1 ura3-1 can1-100 (GAL+) cmk2Δ::Hygro*	This study
EYL5334	*MATa bar1Δ trp1-1 his3-11,15 leu2-3,112 ade2-1 ura3-1 can1-100 (GAL+) ptk2Δ::Hygro*	This study
EYL5335	*MATa bar1Δ trp1-1 his3-11,15 leu2-3,112 ade2-1 ura3-1 can1-100 (GAL+) cnb1Δ::Hygro*	This study
EYL5336	*MATa bar1Δ far1Δ trp1-1 his3Δ200 leu2-3,112 ade2-1 ura3-1 can1-100 (GAL+) FUS1p-HIS3::lys2 pbs2Δ::Hygro*	This study
EYL5337	*MATa bar1Δ far1Δ trp1-1 his3Δ200 leu2-3,112 ade2-1 ura3-1 can1-100 (GAL+) FUS1p-HIS3::lys2 cnb1Δ::Hygro*	This study
EYL5338	*MATa bar1Δ trp1-1 his3-11,15 leu2-3,112 ade2-1 ura3-1 can1-100 (GAL+) kss1Δ::HIS3 fus3Δ::Hygro*	This study
EYL5379	*MATa bar1Δ trp1-1 his3-11,15 leu2-3,112 ade2-1 ura3-1 can1-100 (GAL+) ptk2Δ::Hygro cmk2Δ::Kan*	This study
EYL5380	*MATa bar1Δ trp1-1 his3-11,15 leu2-3,112 ade2-1 ura3-1 can1-100 (GAL+) cmk2-tag(3-GFP)Kan*	This study
EYL5382	*MATa bar1Δ trp1-1 his3-11,15 leu2-3,112 ade2-1 ura3-1 can1-100 (GAL+) crz1Δ::Kan*	This study
S288c deletion strains in Figure 1 are isogenic to S288c BY4741		[46]
BY4741	*MATa his3Δ1 ura3Δ0 leu2Δ0 met15-0*	[47]
RG2468	*ste4Δ::KAN^r ^leu2 ura3 his3 met15*	Research Genetics
RG4038	*ste5Δ::KAN^r ^leu2 ura3 his3 met15*	Research Genetics
RG3439	*ste50Δ::KAN^r ^leu2 ura3 his3 met15*	Research Genetics
RG5271	*ste11Δ::KAN^r ^leu2 ura3 his3 met15*	Research Genetics
RG6981	*kss1*Δ*::KAN^r ^leu2 ura3 his3 met15*	Research Genetics
JAY408	*fus3Δ::KAN^r ^ste5Δ::KAN^r^*	[25]
Isogenic to ∑1278b		
L5528	*ura3-52, his3::hisG*	[48]
L5554	*ste5::LEU2, ura3-52, leu2::hisG*	[48]
Plasmids		
pYGU-11ΔN	*pGAL1-STE11ΔN LEU2 CEN*	[49]
*GAL1 *vector	*pGAL1*, *LEU2 CEN*	Elion lab collection
pYEE133	*pGAL1-STE12 TRP1 CEN*	Elion lab collection
*GAL1 *vector	*pGAL1*, *TRP1 CEN*	Elion lab collection
pOO1	*pCMK2-lacZ, URA3*, 2 μ	[19]
EBL95	*pDL1460 pFUS1::ubiY-lacZ, URA3*, 2 μ	Elion lab collection [50]
EBL183	*PMC1*-*lacZ*, *URA3*, 2 μ	[26]
EBL185	*p*DM5 *FKS2 *(-968-+6)-CYC1-*lacZ*, *URA3*, 2 μ	[26]
EBL201	*FKS2 *(-928-+1)-CYC1-*lacZ*, *URA3*, 2 μ	[14]
EBL200	*FKS2 *(-706-+1)-CYC1-*lacZ*, *URA3*, 2 μ	[14]
EBL1258	*FKS2 *(-540--375)-CYC1-*lacZ*, *URA3*, 2 μ	[15]
EBL513	YKL161c (*MLP1*)-*lacZ*, *URA3*, 2 μ	Elion lab collection
EBL514	YIL117c (*PRM5*)-*lacZ*, *URA3*, 2 μ	[51]
EBL517	pLGΔ-178 pCYC1-*lacZ*, *URA3*, 2 μ	[52]

### Detection of active MAPK in whole cell extracts

Active Slt2p/Mpk1p, Fus3p and Kss1p were detected with anti-phospho-p42p44 antibodies using 200 μg whole cell extract exactly as described [25].

### Galactose induction

Cells were grown at 30°C (unless noted otherwise) to logarithmic phase in medium containing 2% dextrose. Cells were then pelleted and resuspended in medium containing 2% raffinose and 0.1% dextrose and grown to an OD_600 _~ 0.8, then collected and resuspended in medium containing 2% galactose at an OD_600 _~ 0.2, and then grown for another 6 hours.

### β-galactosidase assay

Cells were harvested at 4°C and frozen at -80°C. Pellets were thawed on ice in 0.25 ml breaking buffer (0.1 M Tris-HCl pH 8.0, 20% Glycerol v/v, 1 mM DTT) containing 12.5 μl of 40 mM PMSF (phenyl methyl sulphonylfloride in 95% ethanol). Glass beads were added and samples were vortexed three times at 4°C, added 0.25 ml breaking buffer into tubes and vortexed again. The supernatant was transferred to a new tube, centrifuged at 12,000 g for 15 min at 4°C and clarified supernatant was transferred to a new tube; protein concentration was determined by BIORAD™ assay. A linear range of protein concentration and incubation time was established. Standardized amounts of extracts were mixed with Z buffer (0.06 M Na_2_HPO_4 _**^. ^**7H_2_0, 0.04 M NaH_2_PO_4 _**^. ^**H_2_0, 0.01 M KCl, 0.001 M MgSO_4 _**^. ^**7H_2_O) and incubated for 15 min at 28°C. Reactions were initiated by addition of 0.2 ml ONPG (4 mg/ml) and stopped by addition of 0.5 ml Na_2_CO_3 _(1 M) after the color had changed into light yellow. Read samples at OD_420_, then calculated as nanomoles of ONPG cleaved per min per milligram protein as described [45]. These experiments were performed at least in triplicate from independent yeast transformants.

### RNA preparation for yeast

Cells were grown in the appropriate medium, then 10 ml (OD_600 _~ 0.6-0.8) of cells were collected, washed once with sterile deionized water, and then resuspended in 600 μl TES buffer (10 mM Tris pH 7.5, 10 mM EDTA, 0.5% SDS). Samples were mixed with 500 μl acid washed phenol and incubated 45 min to 1 hour at 65°C with vortexing for 30 sec every 10 min. Samples were then cooled on ice and centrifuged 5 min. and then 0.5 ml aqueous phase was transferred into new tubes and re-extracted with 400 μl acid phenol. These steps were repeated once again. Then 400 μl aqueous phase was transferred into a new tube and mixed with 40 μl sodium acetate (3M, pH 5.3) and 800 μl ethanol and incubated for 1 hour at -20°C. RNA and other precipitated material was washed and the RNA was purified with a Qiagen kit following manufacturer's suggestions. 1-10 μg of RNA was used for cDNA synthesis according to protocol (SuperScript III, Invitrogen) and the cDNA was used for real-time quantitative PCR (RT-qPCR). The following forward and reverse primers were used, respectively: *ACT1*, 5'-TGGATTCCGGTGATGGTGTT-3' and 5'-AAATGGCGTGAGGTAGAGAGAAAC-3'; *FKS2*, 5'- GACTACTGATGAAGATAGAG-3' and 5'- CATGACAAACCCATACAG-3'; *PMC1*, 5'- ACTGTGTGGTATGTTGTC-3' and 5'- TCGAGTCCAAATACGTAC-3'; *PRM5*, 5'- TGGTGTCTACAATCTCTTC-3' and 5'- TGGTTACGATTTACGCTAC-3'.

### Immunoblot analysis

Cmk2p was monitored by western blot analysis of *3xGFP*-tagged and *2xGFP*-tagged Cmk2p. Cells were lysed by vortexing with glass beads in lysis buffer (50 mM Tris-HCl, pH 7.5, 100 mM NaCl, 0.5% triton-X 100, 10 mM NaF, 5 μg/ml aprotinin, 5 μg/ml leupeptin, 0.2 mM Na_3_VO_4_). Cell extracts containing 100 μg of total protein were run on 6% native polyacrylamide gels and then transferred to polyvinylidene difluoride (PVDF) membranes [37]. Membranes were probed with antibody to GFP epitope (Santa Cruz).

### Statistics

Data were expressed as mean ± SE of at least three independent experiments. Statistical significance was computed by the unpaired Student's *t *test. A *p *< 0.05 was considered statistically significant.

## Authors' contributions

XYW helped design and carried out most of the experiments and wrote the manuscript. MAS helped to do the experiments and edited the manuscript. DMS performed experiments and edited the manuscript. EAE conceived the project, supervised experiments and revised the manuscript. All authors read and approved relevant portions of the manuscript.

## Supplementary Material

Additional file 1 **Figure S1. Ste11 *in vitro *phoshorylation links to Cmk2p and Ptk2p overlap other MAPK pathway kinases**. From Ptacek J, Devgan G, Michaud G, Zhu H, Zhu X, Fasolo J, Guo H, Jona G, Breitkreutz A, Sopko R, et al.: Global analysis of protein phosphorylation in yeast. Nature 2005, 438:679-684. Click here for file

Additional file 2 **Figure S2. Level of active Mpk1p and Kss1p in different yeast backgrounds**. This figure shows short and long exposures of immunoblot in Figure 1A to better visualize the relative amount of phosphorylated Mpk1p in W303a compared to S288c. The data in Figure 1A are from a reprobing of the same normalized immunoblot shown in Supplemental Figure Two in Andersson *et al*., 2004 [25]. Click here for file
