# Supplementary figures and images for: Ste11p MEKK signals through HOG, mating, calcineurin and PKC pathways to regulate the FKS2 gene
Source: BMC Mol Biol. 2011 Nov 24;12:51. doi: 10.1186/1471-2199-12-51 (PMC3233502; doi:10.1186/1471-2199-12-51)

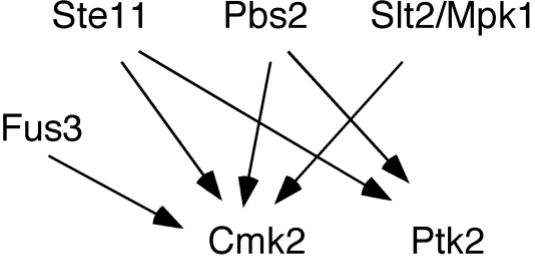

Phosphorylation links  
from Ptacek *et al*, 2005

Supplement: Additional file 1 — Figure S1. Ste11 in vitro phoshorylation links to Cmk2p and Ptk2p overlap other MAPK pathway kinases. From Ptacek J, Devgan G, Michaud G, Zhu H, Zhu X, Fasolo J, Guo H, Jona G, Breitkreutz A, Sopko R, et al.: Global analysis of protein phosphorylation in yeast. Nature 2005, 438:679-684. [file 1471-2199-12-51-S1.PDF]

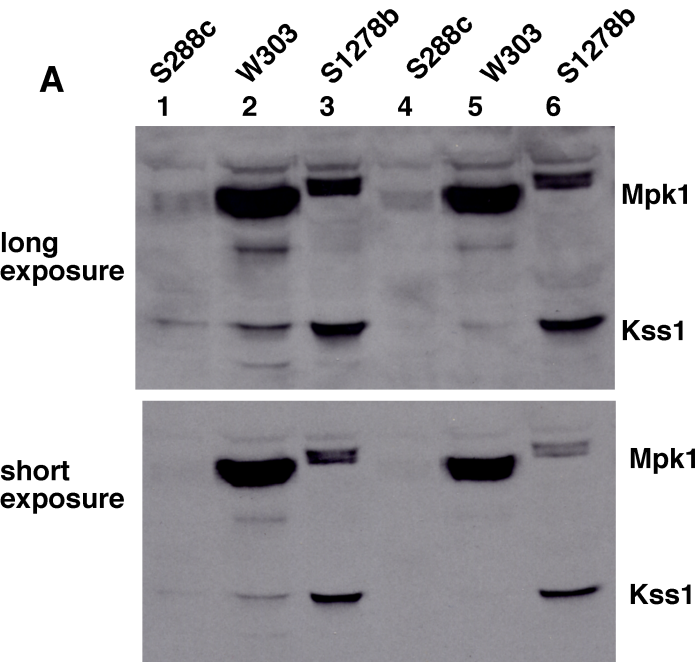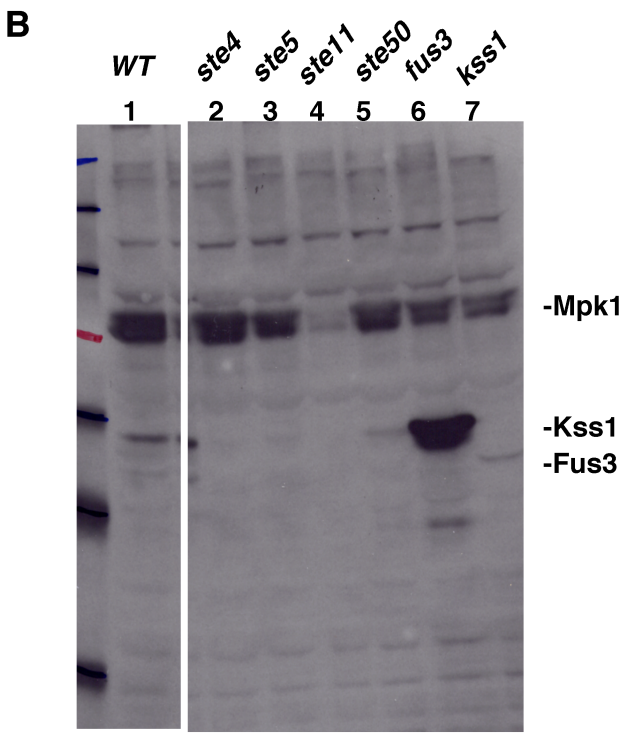

Supplement: Additional file 2 — Figure S2. Level of active Mpk1p and Kss1p in different yeast backgrounds. This figure shows short and long exposures of immunoblot in Figure 1A to better visualize the relative amount of phosphorylated Mpk1p in W303a compared to S288c. The data in Figure 1A are from a reprobing of the same normalized immunoblot shown in Supplemental Figure Two in Andersson et al., 2004 [25]. [file 1471-2199-12-51-S2.PDF]
